# Atrial Fibrillation in Heart Failure with Preserved Left Ventricular Systolic Function: Distinct Elevated Risk for Cardiovascular Outcomes in Women Compared to Men

**DOI:** 10.3390/jcdd9120417

**Published:** 2022-11-26

**Authors:** Alaa Mabrouk Salem Omar, Mohamed Ahmed Abdel Rahman, Osama Rifaie, Jonathan N. Bella

**Affiliations:** 1 Department of Cardiology, Mount Sinai Morningside, New York, NY 10025, USA; 2 Department of Cardiology, Icahn School of Medicine at Mount Sinai, New York, NY 10029, USA; 3 Department of Cardiology, Ain Shams University, Cairo 4393002, Egypt; 4 Department of Cardiology, BronxCare Health System, Bronx, NY 10457, USA

**Keywords:** atrial fibrillation, heart failure with preserved ejection fraction, women

## Abstract

**Background:** Heart failure with preserved ejection fraction (HFpEF) is prevalent in women and is associated with atrial fibrillation (AF). However, sex associations in AF-related HFpEF are not well explored. **Aim:** We studied differences between men and women with and without AF-related HFpEF symptoms on left ventricular (LV) geometry and diastolic dysfunction (DD) and their effect on cardiovascular events. **Methods:** Retrospectively, HFpEF patients with and without a history of AF referred for echocardiography were studied. Echocardiographic assessments were focused on LV geometry and diastolic functions. Patients were followed for the occurrence of cardiac events defined as death and cardiac hospitalization. **Results:** We studied 556 patients [age: 66.7 ± 17 years, 320 (58%) women, 91 (16%) AF]. Compared to HFpEF without AF (HFpEF-AF), HFpEF with AF patients (HFpEF+AF) were older (76 ± 13.8 vs. 64.9 ± 17.3 years, *p* < 0.001), had more risk factors, comorbidities, left ventricular hypertrophy (32 vs. 13%, *p* < 0.001), higher relative wall thickness (0.50 ± 0.14 vs. 0.44 ± 0.15, *p* < 0.001), and DD (56 vs. 30%, all *p* < 0.001). HFpEF+AF women had the worst clinical, LV geometric, and diastolic functional profiles and highest rates of cardiovascular outcomes compared to HFpEF+AF men and were the only group to predict outcomes (HR: 2.7, 95%CI: 1.4–5.1), while HFpEF-AF women were a low-risk group; HFpEF+AF and HFpEF-AF men had intermediate cardiovascular outcomes which were confirmed after propensity score matching. **Conclusions:** Among patients with HFpEF, women with AF had more abnormal LV geometry and diastolic function and had an increased risk of adverse cardiovascular outcomes independent of traditional risk factors, comorbidities, and baseline diastolic function.

## 1. Introduction

Heart failure with preserved ejection fraction (HFpEF) is a clinical syndrome that is associated with at least comparable morbidity and mortality to patients with heart failure with reduced ejection fraction (HFrEF). Despite the overlapping prognosis and clinical presentation, most of the medications used in HFrEF are not associated with clinical benefit in HFpEF, and the only medications that are associated with any level of clinical benefit do so modestly or only for symptom management. Risk factors and clinical phenotypes of HFpEF are significantly heterogeneous; however, atrial fibrillation (AF) has been linked to a specific high likelihood of development of left atrial enlargement, the elevation of left ventricular (LV) filling pressure (LVFP), diastolic dysfunction, subsequent development of HFpEF, and increased association with adverse cardiac outcomes. 

Epidemiologically, HFpEF is more prevalent in women than men, with nearly a 2:1 ratio. In randomized clinical trials concerned with heart failure, men represent the majority of the studies concerned with HFrEF, while the representation in randomized clinical trials in patients with HFpEF seems to be mostly by women. The PARAGON-HF study increased the interest in the sex-specific differences in HFpEF when it was found that the only group of HFpEF patients that showed clinically meaningful response to sacubitril/valsartan therapy was women; however, the reasons behind that are not yet explored [1]. 

Recent studies suggest that women with HFpEF carry higher clinical risks and an increased incidence of diastolic dysfunction when compared with men. Conversely, however, AF as a strong predictor of HFpEF is more prevalent in men. The sex-related differences in AF-related HFpEF and its clinical prognosis is less explored [2]. Along these lines, the current study aimed at exploring the differences between men and women with and without AF-related HFpEF symptoms and their effect on LV geometrical and diastolic function properties and adverse cardiovascular events.

## 2. Methods

In a retrospective study, patients with an established diagnosis of HFpEF referred for echocardiography on an outpatient basis in our echocardiography laboratories in the period between January 2015 and December 2017 were included. Patients of both sexes were included and excluded if they had EF < 50% if they had more than moderate valve disease, and if the echocardiographic assessments were not technically sufficient or when images were of poor quality. Echocardiographic assessments were focused on LV geometrical assessments and assessment of diastolic function, and history was focused on identifying patients with a history of atrial fibrillation. All authors had full access to the data, and the study research protocol was approved by the institutional review board. 

### 2.1. Echocardiographic Analyses

All patients underwent echocardiographic recordings obtained with commercially available systems (GE Vivid 7 and 9, GE Healthcare, Wauwatosa, WI). Digital routine grayscale 2-dimensional and tissue Doppler recordings were obtained from 3 consecutive beats at end-expiration from standard apical views at depths of 12–20 cm. Left ventricular ejection fraction (LV-EF) and left atrial volume indexed to body surface area (LAVi) were calculated using the modified Simpson’s technique from the apical 2- and 4-chamber images. Left ventricular geometry was assessed by calculating relative wall thickness (RWT) and LV mass index (LVMi). RWT was calculated using the recommended formula: 

RWT = 2 × PWd/LVEDD, where PWd is the posterior wall dimension, and LVEDD is the left ventricular end-diastolic dimension. LVMi was calculated using the formula LVMi = [0.8 × (1.04 × (((LVEDD + IVSd +PWd)^3^ − LVEDD^3^))) + 0.6]/BSA, where LVEDD is left ventricular end-diastolic dimension, IVSd is the interventricular septal dimension, and PWd is posterior wall dimension. All geometrical measurements were done at end-diastole from the parasternal long-axis window 1 mm below the mitral valve tips

The pulsed-wave Doppler-derived transmittal velocity and spectral tissue Doppler-derived mitral annular velocity were obtained from the apical 4-chamber view. The early diastolic wave velocity (E) and the late diastolic atrial contraction wave velocity (A) were measured using pulsed-wave Doppler recording, and the early diastolic mitral annular velocity (e’) was measured from the septal mitral annular positions. The E/e’ ratio was calculated to assess LV filling pressure (LVFP) for all patients. In addition, the peak tricuspid valve regurgitation velocity was measured, and the presence of diastolic dysfunction was assessed as recommended by the American Society of Echocardiography guidelines. All measurements were made in ≥3 consecutive cardiac cycles, and average values were used for the final analyses.

### 2.2. Study Endpoints

Patients were followed for a median of 1.1 years (13 months) for cardiovascular death or hospitalization for cardiovascular causes. Events were recorded by chart review and by means of telephone call contact with patients. Patients were checked for the prediction of outcomes based on their sex, presence of a history of AF or elevated LVFP at the time of the study. 

### 2.3. Statistical Methods

Categorical data are presented as numbers (%) and were compared using the chi-square test. Continuous data are presented as mean ± SD. Data were tested for normality using Kolmogorov-Smirnov and Shapiro-Wilk tests and, accordingly, continuous data were compared using a *t*-test or analysis of variance (ANOVA) if they are normally distributed or the Mann–Whitney U test if they are not normally distributed. Cox regression and Kaplan–Meir survival curves were used to assess the predictability of outcomes in different subgroups. 

A 1:1 propensity score matching was conducted to match patients with AF to patients without AF. The propensity score was done using binominal logistic regression with the independent variables (covariates) being age, risk factors (diabetes, hypertension), presence of co-morbidities (anemia, defined by hemoglobin level, renal dysfunction defined by creatinine, and presence of chronic obstructive pulmonary disease), and absolute parameters of diastolic functions (LAVi, E/A ratio, e’ velocity from the septal mitral annular side, E/e’ ratio, and tricuspid regurgitation velocity). 

A 1:1 propensity score matching was done using the nearest neighbor classification and resulted in 156 matched patients (78 in each group)

Differences were considered statistically significant at *p* < 0.05. All analyses will be performed with commercially available software (SPSS, version 23.0; SPSS, Inc., Chicago, IL, USA.).

## 3. Results

During the specified period, 556 patients were retrospectively studied. Table 1 summarizes the baseline demographic, clinical, laboratory, as well as echocardiographic data of the study population. The mean age was 66.7 ± 17 years, and 320 (58%) patients were women. Moreover, 91 (16%) patients had AF, of whom 18 (20%) patients had permanent AF (age: 75.4 ± 14 years, 14 women, EF: 63 ± 6%), 37(41%) had persistent AF (age: 76 ± 14 years, 14 women, EF: 63 ± 5%), and 36 (39%) had paroxysmal AF (age: 76 ± 14 years, 11 women, EF: 63 ± 6%). There was no difference between different types of AF regarding age, sex, risk factors, or LV functions. Importantly, 54 (59%) patients were on anticoagulation at the time of the study, and 37 (41%) patients were not on anticoagulation due to high bleeding risk or because patients refused anticoagulation.

Compared to HFpEF men, HFpEF women showed no statistically significant difference in age, or cardiac risk factors, including a history of diabetes mellitus, hypertension, and hyperlipidemia, except for more smoking in men. Regarding comorbidities, more men had chronic obstructive pulmonary disease (COPD) and worse renal functions, as suggested by serum creatinine, while women were slightly more anemic, as suggested by hemoglobin levels.

Compared to HFpEF patients without AF (HFpEF-AF), HFpEF patients with AF (HFpEF+AF) were older and had more cardiac risk factors except for similar history of diabetes. Regarding comorbidities, HFpEF+AF patients had worse renal function and were more anemic but had a similar history of COPD compared to HFpEF-AF patients.

### 3.1. Echocardiographic Comparisons

The overall and subgroup description of echocardiographic comparisons of structural and functional characteristics are summarized in Table 1. Briefly, the mean left ventricular ejection fraction (EF) was 63.1 ± 5.8. In terms of diastolic function, 244 patients of the study group had normal diastolic function, 191 patients had evidence of diastolic dysfunction, and 121 patients were indeterminate.

Compared to HFpEF men, HFpEF women were found to have higher EF values, lower LV end-diastolic volume index (LVEDVi), lower LV end-systolic volume index (LVESVi), similar LAVi, and relative wall thickness (RWT), lower left ventricular mass indexed to body surface area (LVMi) but similar prevalent LV hypertrophy. HFpEF women were found to have similar absolute diastolic variables compared to HFpEF men, namely E-wave velocity, e’ velocity, E/e’ ratio, and TRV velocity, with the exception of higher A wave velocity translating into lower E/A ratio, and a shorter E-wave deceleration time. The occurrence of diastolic dysfunction was also not different between men and women.

On the other hand, compared to HFpEF-AF patients, patients with AF were found to have similar EF, LVEDVi and LVESVi; however, they had higher LAVi, RWT, and LVMi. Diastolic parameters were statistically significantly worse in HFpEF+AF compared to HFpEF-AF, namely higher E-wave velocity, higher E/A ratio, lower e’ velocity, higher E/e’ ratio, and higher TRV velocity, except for similar A wave velocity and E-wave deceleration time. Diastolic dysfunction was diagnosed significantly more in HFpEF+AF.

### 3.2. Classifications and Comparisons Based on Sex and Rhythm

The demographic, clinical, and echocardiographic comparisons of HFpEF women and men with and without AF are summarized in Table 2.

Briefly, age, cardiac risk factors, comorbidities (COPD, anemia and renal dysfunction), EF, LAVi, and echocardiographic measures of geometry and diastolic function were significantly different between sexes with and without AF. HFpEF-AF women represented the lowest risk profile among all these parameters. Furthermore, HFpEF+AF women had the worst demographic and clinical profiles in terms of being older, having the most history of hypertension and hyperlipidemia, having the highest prevalence of COPD, having worse renal function, and having lower hemoglobin levels. In terms of echocardiographic parameters, HFpEF+AF women were found to have the smallest LVEDVi and LVESVi, the largest LAVi, the worst RWT and LVMi, the lowest septal e’ velocity and E/e’ ratio, the highest TRV velocity, and higher prevalent diastolic dysfunction than HFpEF+AF men. HFpEF+AF men had a higher history of diabetes and smoking, worse renal function, higher E-wave velocity and E/A ratio and shorter E-deceleration time while sharing a similar high-risk profile with HFpEF+AF women with regard to a history of COPD, hemoglobin levels, and E/e’ ratio.

### 3.3. Outcomes

In the study follow-up period, 25 patients died, 91 patients were hospitalized for cardiac causes, and 99 patients had the composite of death or cardiac hospitalization (Table 1). There was no significant difference between HFpEF men and women regarding individual or composite outcomes. While HFpEF-AF patients had similar death rates compared to HFpEF+AF patients (*p* = 0.291; Table 1), more cardiac hospitalization and composite outcomes occurred among HFpEF+AF patients (*p* = 0.012, 0.008).

HFpEF+AF women had the highest rates of death, cardiac hospitalization, and composite outcomes (*p* = 0.036, 0.018 and 0.014, all *p* < 0.05, respectively; Table 2), while HFpEF-AF women had the lowest rates with both HFpEF+AF and HFpEF-AF men had intermediate outcomes (Table 2).

**Figure 1:**
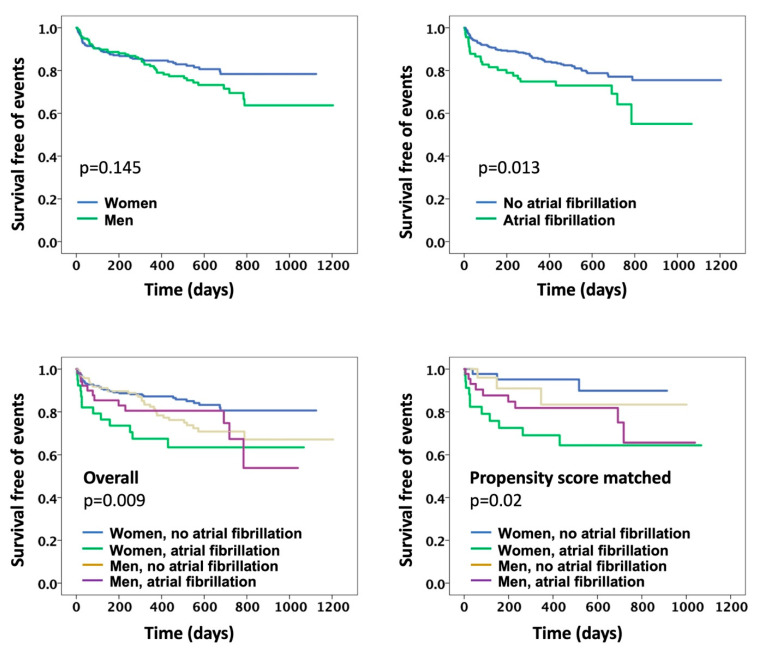
Kaplan-Meir survival curves for composite outcomes in the study. The Upper left panel shows the comparison between men and women, showing no significant difference. The upper right panel shows the comparison between patients with and without atrial fibrillation (AF), showing an increased risk in patients with AF. The lower right panel shows the comparison of subgroups classified based on sex and presence of AF showing women with AF to be the highest risk category. The lower left panel shows the comparison of subgroups classified based on sex and presence of AF after 1:1 propensity score matching for co-variates showing women with AF to persist as the highest risk category.

Cox-regression models revealed that female sex was not predictive of death (HR: 1.17, 95%CI: 0.53–2.6; Table 3, Figure 1), cardiac hospitalization (HR: 1.35, 95%CI: 0.88–2.1, Table 3, Figure 1), or composite outcomes (HR: 1.3, 95%CI: 0.9–2). AF alone was not predictive of death (HR: 1.63, 95%CI: 0.65–4.1; Table 3, Figure 1) but was associated with cardiac hospitalization (HR: 1.7, 95%CI: 1.02–2.8), and the composite outcomes (HR: 1.77, 95%CI: 1.1–2.8; Table 3, Figure 1).

In the subgroup analyses, it was found that HFpEF+AF women were the only group to significantly predict death (HR: 4.3, 95%CI: 1.5–12.9; Table 3, Figure 1), cardiac hospitalization (HR: 2.8, 95%CI: 1.4–5.6; Table 3, Figure 1), and composite outcomes (HR: 2.7, 95%CI: 1.4–5.1; Table 3, Figure 1).

### 3.4. Propensity Score Matching Comparisons

Patients with and without atrial fibrillation were matched for all covariates based on a 1:1 propensity score matching model as previously described. The model yielded 156 matched patients (78 in each group; Table 4). As can be seen in Table 4, patients with and without AF after matching were similar in all clinical, demographic, comorbidities, EF, and echocardiographic parameters geometry and diastolic functions.

In the matching group, it was found that HFpEF+AF women remained the only group associated with death (HR: 7, 95% CI: 0.81–60; Table 3, Figure 1), cardiac hospitalization (HR: 4.96, 95% CI: 1.05–23.6, Table 3; Figure 1) and composite outcomes (HR: 5.5, 95% CI: 1.5–20, Table 3; Figure 1).

## 4. Discussion

The main findings of our study are the following: first, in a consecutive group of patients with HFpEF symptoms, the majority of HFpEF patients (58%) were women, and 16% of the overall group had a history of AF. Second, while HFpEF women did not differ compared to men in the overall clinical or demographic parameters and did differ in adverse cardiac events, AF was more common in HFpEF men and was associated with worse demographic, clinical and echocardiographic measures of geometry and diastolic function and worse adverse cardiovascular events. Finally, HFpEF+AF women had worse clinical, LV geometric and diastolic functional profiles and were associated with a higher risk of adverse events, while HFpEF-AF women were a low-risk group, and HFpEF+AF and HFpEF-AF men were the intermediate groups.

### 4.1. Women in Heart Failure Preserved Ejection Fraction

Epidemiological studies suggest that women predominate in the incidence of HFpEF with a women-to-men ratio that approximates 2:1 [3]. This women-predominant pattern is the strongest distinguishing feature of HFpEF from HFrEF, and, similar to our study where women accounted for 58% of all study subjects, clinical trials testing effects of therapeutics in both conditions have shown a similar distribution of women among study participants (20–25% in HFrEF clinical trials and 50–60% in HFpEF clinical trials) [1,4,5].

HFpEF is known to be associated with changes in ventricular geometrical properties and cardiac remodeling that become more pronounced with aging, in hypertensives, as well as with AF [6]. The result would lead to diastolic dysfunction and/or elevated LVFP and, eventually, systolic dysfunction. Reportedly, such structural and hemodynamic alterations that occur with HFpEF are observed more in women when compared with men. As shown in previous reports, maladaptive changes in LV geometry, such as LV hypertrophy and increased ventricular stiffness, are more dramatic in women when compared to men [3]. In our study, compared to men, geometrical differences were evident in women who had smaller LV and higher ejection fraction; however, this did not translate into differences in LV thickness or mass or differences in diastolic functions. We believe that the reasons, in general, were that men were more likely smokers, had higher rates of comorbidities such as COPD and renal dysfunction, and had more AF, which may have offset the general differences observed between men and women regarding maladaptive structural responses. A closer look, however, shows signals that point out a slightly worse diastolic function in women still existed compared to men, specifically with mitral flow parameters such as E-deceleration time and the tricuspid regurgitation velocity.

According to recent studies, there are no overall differences in clinical outcomes between HFpEF women and men. In our study as well, the overall risk of outcomes was not different between men and women. It is to be highlighted that, in some studies, women with HFpEF carry a higher risk of outcomes after adjustment of covariates [6], suggesting that women’s chance for the development of adverse events follows an untraditional risk profile.

### 4.2. Atrial Fibrillation in Women with Heart Failure with a Preserved Ejection Fraction

Epidemiologically, there is a strong relationship between HFpEF and AF. The relationship seems to be bidirectional, where patients with AF have an elevated risk of developing HFpEF and, conversely, most patients with HFpEF eventually develop AF [7]. Such HFpEF/AF relationship is still underestimated as subclinical episodes of AF can occur for years. No matter the stage of presentation, patients with incidentally discovered AF have increased LVFP and larger left atrial size and volumes [8]. The relationship of AF and HFpEF is so strikingly parallel that it is a matter of “the chicken or the egg” type of dilemma.

In our study, we confirmed such a high-risk association when we found that the presence of a history of AF is a very strong predictor of clinical, geometrical, and hemodynamic alteration in HFpEF patients, as well as a predictor of LV diastolic functions. Moreover, AF was a significant predictor of adverse cardiac events.

In our study AF was more prevalent in men compared to women (relative risk 1.81, 95% CI: 1.23–2.64, *p* = 0.002), a finding that is in agreement with prior reports that confirm a 1.5 to 2 increased risk of AF in men compared to women [9]. Surprisingly, however, when groups were classified based on sex and the presence of AF, the co-existence of AF in HFpEF women and not in HFpEF men was associated with worse clinical profiles, LV geometry, LV diastolic dysfunction, and cardiovascular events.

A number of observational studies have demonstrated the association between female sex and the risk of AF-related stroke and thromboembolism [10]. However, the relationship between AF and heart failure presentation and outcomes in women is less established. Most of the available evidence concerned with AF-related heart failure risk points towards no difference between men and women; however, a few studies have suggested higher risk in women [9,11,12]. Moreover, several studies have evaluated the interaction between sex and AF-related risk of mortality, but the results have not been consistent [13,14,15,16]. The inconsistencies in the AF relationships with heart failure in women are probably related to several factors, such as these studies were mainly derived from HFrEF and that there is scarce data in patients with HFpEF in addition to the lack of dedicated studies that assess the risk in women.

### 4.3. Clinical and Mechanistic Implications and Future Directions

While traditional risk factors still affect myocardial function similarly in women and men, women exhibit sex-specific physiological and pathological elements beyond traditional risk factors that may independently cause differences in progression, clinical presentation, and outcomes of myocardial dysfunction, as well as the development of AF. Some of these women-specific risk factors include the lack of protective estrogen in the perimenopausal period or estrogen receptor dysfunction, pregnancy, breast cancer-related treatments, specific responses to psychological stressors and a greater prevalence of autoimmune diseases [17].

Such sex-specific elements can be linked to differences in the effects of therapeutics between women and men, such as that observed in the PARAGON-HF trial [18]. Moreover, such risk women-specific factors can play a distinct role in the development as well as the outcomes of AF.

Despite the fact that women a smaller left atria and ventricles compared to men, as shown in our study, small magnetic resonance studies have shown that atrial fibrosis in patients with HFpEF was detected in women more than in men [19] and that plasma levels of inflammatory markers such as C-reactive protein [20] and fibroblast growth factor [21] are higher in women than in men suggesting an increased risk of AF.

It’s important here to note that, despite that such effects are recognized in the literature, they are mostly driven by retrospective and post hoc sex-based analyses. The translation into clinical practice is limited by the underrepresentation of women in clinical trials and the lack of sex-specific prospective studies.

### 4.4. Study Limitations

This is a retrospective study with a relatively small number of patients, and larger prospective studies will need to be done to confirm the study findings. The plasma levels of brain natriuretic peptide were not measured in this study as patients were assessed on an outpatient basis and should be measured to detect differences in future studies. In our study, there was no assessment of the left atrial anatomic substrates and fibrosis and no assessment of LA or LV mechanical behavior, such as strain and strain rate measurements which could further elucidate differences between men and women. Moreover, heart rate is a known parameter to affect echocardiographic measurements in patients with atrial fibrillation, but heart rate data was not available in our study. However, in our laboratory, and as recommended by the American society of echocardiography, measurements in patients with atrial fibrillation are averaged from at least three cardiac cycles. Finally, despite the rigorous matching done for clinical and echocardiographic covariates, there is a possibility the variables unaccounted for that affect clinical outcomes and diastolic properties may influence the predictive ability of our models. As such, the models used in our study were done for qualitative exploratory purposes and model validation was not attempted. Therefore, future prospective analyses should be performed to confirm our findings and validate the models used.

## 5. Conclusions

AF carries a special risk for the development of outcomes in patients with HFpEF. Despite the fact that AF is more common in men, women with AF are a special group of patients with worse clinical, left ventricular geometrical, and diastolic profiles and were the only group in our study to be associated with adverse clinical outcomes. As such, women with AF-associated HFpEF are at a particular increase of adverse cardiovascular outcomes independent of traditional risk factors, comorbidities and baseline diastolic function, a finding that seems more pronounced when compared to men. Further studies are needed to determine the underlying pathophysiology and to identify novel preventive and disease-modifying treatments for HFpEF+AF.

## Figures and Tables

**Table 1:** Demographic, clinical and echocardiographic data in all patients as well as in groups.

	All Patients (*n* = 556)	Women (*n* = 320)	Men (*n* = 236)	*p*-Value	No AF (*n* = 465)	AF (*n* = 91)	*p*-Value
Age, years *	66.7 ± 17	66 ± 17.4	67.7 ± 17	0.262	64.9 ± 17.3	76 ± 13.8	<0.001
Women, *n* (%)	320 (58)	320 (100)	0 (0)	<0.001	281 (62)	31 (34)	<0.001
Diabetes, *n* (%) *	131 (24)	74 (23)	57 (24)	0.778	106 (23)	25 (27)	0.336
Hypertension, *n* (%) *	323 (58)	186 (58)	137 (58)	0.986	256 (55)	67 (74)	0.001
Smoking (current/ex), *n* (%)	228 (41)/35 (6)	103 (32)/12 (4)	125 (53)/23 (10)	<0.001	178 (38)/30 (6)	50 (55)/5 (5)	0.014
Hyperlipidemia, *n* (%)	261 (47)	150 (47)	111 (47)	0.970	206 (44)	55 (60)	0.005
Atrial fibrillation, *n* (%)	91 (16)	39 (12)	52 (22)	0.002	0 (0)	91 (100)	<0.001
Chronic Obstructive pulmonary disease, *n* (%) *	43 (8)	18 (6)	25 (11)	0.03	32 (7)	11 (12)	0.09
Hemoglobin, g/dL*	11.7 ± 2.1	11.6 ± 1.8	11.9 ± 2.4	0.06	11.9 ± 2	11.2 ± 2.1	0.004
Creatinine, mg/dL*	1.17 ± 0.97	1 ± 0.9	1.3 ± 1	<0.001	1.1 ± 0.9	1.4 ± 1.3	0.008
LV ejection fraction, (%)	63.1 ± 5.8	64.2 ± 5.6	61.6 ± 5.7	<0.001	63.2 ± 5.8	62.9 ± 5.5	0.702
LV end diastolic volume index, mL/m^2^	46.9 ± 15	44.3 ± 14	50.2 ± 17	<0.001	46.8 ± 15	47.5 ± 17	0.760
LV end-systolic volume index, mL/m^2^	17.7 ± 7	16.2 ± 6	19.6 ± 8	<0.001	17.6 ± 7	17.9 ± 7	0.795
Left atrial volume index, mL/m^2^ *	37.3 ± 14.7	36.9 ± 15.1	37.7 ± 14.2	0.541	35.1 ± 12.8	47.9 ± 18.9	<0.001
Relative wall thickness	0.45 ± 0.15	0.46 ± 0.17	0.45 ± 0.12	0.880	0.44 ± 0.15	0.5 ± 0.14	0.001
LV mass index, g/m^2^	77.8 ± 29	73.2 ± 26.7	83.9 ± 31.7	<0.001	74.2 ± 26.2	96 ± 37.2	<0.001
Abnormal LV mass index	91 (16)	59 (18)	32 (14)	0.124	62 (13)	29 (32)	<0.001
E-wave velocity, cm/s	81.6 ± 25.3	82.2 ± 23.9	80.8 ± 27.3	0.509	78.8 ± 22.5	96 ± 33	<0.001
A-wave velocity, cm/s	85.5 ± 32	88.7 ± 30.8	81 ± 33.4	0.005	84 ± 31	90 ± 36	0.105
E/A *	1.07 ± 0.53	1.02 ± 0.46	1.13 ± 0.6	0.014	1 ± 0.47	1.25 ± 0.76	<0.001
E-DcT, ms	269 ± 89	258 ± 82.6	285 ± 95.7	<0.001	269 ± 89	269 ± 89	0.996
E’ velocity septal, cm/s *	6.3 ± 3.2	6.3 ± 3.7	6.2 ± 2.24	0.522	6.4 ± 3.3	5.4 ± 2	0.003
E/e’ ratio septal *	15.6 ± 9.4	15.8 ± 9.5	15.3 ± 9.3	0.503	14.5 ± 8.2	21.2 ± 12.6	<0.001
E/e’ ≥ 14	244 (44)	144 (45)	100 (42)	0.537	185 (40)	59 (65)	<0.001
Tricuspid regurgitation velocity, m/s *	2.45 ± 0.86	2.5 ± 1.05	2.4 ± 0.48	0.08	2.4 ± 0.9	2.6 ± 0.5	0.045
Diastolic dysfunction (No/Yes/Indeterminate), *n* (%)	244 (44)/191 (34)/121 (22)	144 (45)/109 (34)/67 (21)	100 (42)/82 (35)/54 (23)	0.791	217 (47)/140 (30)/108 (23)	27 (30)/51 (56)/13 (14)	<0.001
Death, *n* (%)	25 (4)	14 (4)	11 (5)	0.872	19 (4)	6 (7)	0.291
Cardiac Rehospitalization, *n* (%)	91 (16)	45 (14)	46 (19)	0.09	68 (15)	23 (25)	0.012
Composite, *n* (%)	99 (18)	52 (16)	47 (20)	0.264	74 (16)	25 (27)	0.008

* Parameters used for 1:1 propensity score matching. The propensity score was calculated for matching of clinical parameters known to affect survival risk (age, diabetes, hypertension) as well as co-morbidities (anemia, defined by hemoglobin level, renal dysfunction defined by creatinine, and presence of chronic obstructive pulmonary disease), and absolute parameters of diastolic functions, namely left atrial volume index (LAVi), E/A ratio, e’ velocity from the septal mitral annular side, E/e’ ratio, and tricuspid regurgitation velocity.

**Table 2:** Demographic, clinical and echocardiographic data in subgroups classified based on sex and presence of atrial fibrillation.

	Women without AF (*n* = 281)	Women with AF (*n* = 39)	Men without AF (*n* = 184)	Men with AF (*n* = 52)	*p*-Value
Age, years	64.6 ± 17.3	76.1 ± 14.8	65.3 ± 17.3	75.9 ± 13.1	<0.001 *#$§
Diabetes, *n* (%)	68 (24)	6 (15)	38 (21)	19 (37)	0.065
Hypertension, *n* (%)	156 (56)	30 (77)	100 (54)	37 (71)	0.011
Smoking (current/ex), *n* (%)	89 (32)/11 (4)	14 (36)/1 (3)	89 (48)/19 (10)	36 (69)/4 (8)	<0.001
Hyperlipidemia, *n* (%)	124 (44)	26 (67)	82 (45)	29 (56)	0.029
Chronic Obstructive pulmonary disease, *n* (%)	13 (5)	5 (13)	19 (10)	6 (12)	0.046
Hemoglobin, g/dL	11.7 ± 1.7	11.3 ± 2	12.1 ± 2.4	11.1 ± 2.2	0.001 @§
Creatinine, mg/dL	1.0 ± 0.78	1.3 ± 1.5	1.3 ± 1.0	1.5 ± 1.1	<0.001 @#
LV ejection fraction, (%)	64.2 ± 5.6	64.3 ± 5.1	61.5 ± 5.8	61.8 ± 5.6	<0.001 @#$
LV end-diastolic volume index, mL/m^2^	44.4 ± 14	43.5 ± 12	50.1 ± 16	50.5 ± 20	0.003@#
LV end-systolic volume index, mL/m^2^	16.2 ± 6	16.1 ± 5	19.7 ± 8	19.3 ± 8	<0.001@#$
Left atrial volume index, mL/m^2^	35.2 ± 13.5	49.4 ± 20	35.1 ± 11.7	46.8 ± 18.3	<0.001 *#$§
Relative wall thickness	0.45 ± 0.17	0.52 ± 0.14	0.44 ± 0.11	0.49 ± 0.13	0.006 *$
LV mass index, g/m^2^	71.1 ± 25.9	88.9 ± 27.53	79 ± 26.1	101.3 ± 42.5	<0.001 *@#§
Abnormal LV mass index	45 (16)	14 (36)	17 (9)	15 (29)	<0.001
E-wave velocity, cm/s	81.1 ± 22.1	90.7 ± 33.7	75.2 ± 22.8	100.7 ± 32.4	<0.001 *$¶§
A-wave velocity, cm/s	88.3 ± 30.2	91.9 ± 34.5	78.6 ± 31.4	89.3 ± 38.9	0.004 @
E/A	1.01 ± 0.44	1.10 ± 0.59	1.07 ± 0.50	1.37 ± 0.85	<0.001 #§
E-DcT, ms	255 ± 80	273 ± 99	290 ± 99	266 ± 82	0.001 @
E’ velocity septal, cm/s	6.5 ± 3.9	5.1 ± 1.9	6.3 ± 2.2	5.5 ± 2.1	0.027 *
E/e’ ratio septal	15.1 ± 8.7	21 ± 13	13.5 + 7.2	21.5 ± 12.6	<0.001 *#$§
E/e’ ≥ 14	2.47 ± 1.1	2.69 ± 0.58	2.35 ± 0.48	2.57 ± 0.46	0.075
Tricuspid regurgitation velocity, m/s	132 (47)/85 (30)/64 (23)	12 (31)/24 (62)/3 (8)	85 (46)/55 (30)/44 (24)	15 (29)/27 (52)/10 (19)	<0.001
Diastolic dysfunction (No/Yes/Indeterminate), *n* (%)	9 (3)	5 (13)	10 (5)	1 (2)	0.036
Death, *n* (%)	34 (12)	11 (28)	34 (18)	12 (23)	0.018
Cardiac Rehospitalization, *n* (%)	39 (14)	13 (33)	35 (19)	12 (23)	0.014

* *p* < 0.05 between women without AF and women with AF, @, *p* < 0.05 between women without AF and men without AF, #, *p*< 0.05 between women without AF and men with AF, $, *p* < 0.05, between Women with AF and men without AF, ¶, *p* < 0.05 between women with AF and men with AF, §, *p* < 0.05, between men without AF and men with AF.

**Table 3:** Cox regression analysis for the prediction of outcomes.

	Death			Cardiac Hospitalization			Composite		
	HR	95%CI	*p*-Value	HR	95%CI	*p*-Value	HR	95%CI	*p*-Value
Women	1.2	0.5–2.6	0.704	1.4	0.9–2.1	0.170	1.3	0.9–2.0	0.146
Atrial fibrillation	1.6	0.7–4.1	0.298	1.7	1.0–2.8	0.042	1.8	1.1–2.8	0.014
Subgroups *									
Women with atrial fibrillation	4.3	1.5–12.9	0.009	2.8	1.4–5.6	0.005	2.7	1.4–5.1	0.002
Men without atrial fibrillation	1.9	0.8–4.7	0.159	1.6	0.9–2.6	0.07	1.6	1–2.4	0.06
Men with atrial fibrillation	0.6	0.1–4.9	0.653	1.6	0.8–3.3	0.185	1.7	0.9–3.3	0.100
Subgroups in matched groups *#									
Women with atrial fibrillation	7	0.8–60.0	0.076	5.0	1.1–23.6	0.044	5.5	1.5–20.0	0.008
Men without atrial fibrillation	1.6	0.1–26.0	0.728	1.9	0.3–11.4	0.493	1.8	0.4–8.8	0.484
Men with atrial fibrillation	1.1	0.1–17.0	0.961	3.2	0.7–15.4	0.149	3.3	0.9–3.3	0.08

* hazard ratios in the subgroups were computed compared to women without atrial fibrillation. # matching was done based on a 1:1 propensity score matching model for age, risk factors (diabetes, hypertension), presence of co-morbidities (anemia, defined by hemoglobin level, renal dysfunction defined by creatinine, and presence of chronic obstructive pulmonary disease), and absolute parameters of diastolic functions.

**Table 4:** Demographic, clinical and echocardiographic data in women and men after 1:1 propensity score matching.

	No AF (*n* = 78)	AF (*n* = 78)	*p*-Value
Age, years	75.5 ± 12.3	74.9 ± 14.3	0.783
Women, *n* (%)	50 (64)	34 (44)	0.01
Diabetes, *n* (%)	17 (22)	22 (28)	0.355
Hypertension, *n* (%)	56 (72)	54 (69)	0.725
Smoking (current/ex), *n* (%)	33 (42)/5 (6)	41 (53)/3 (4)	0.396
Hyperlipidemia, *n* (%)	46 (59)	43 (55)	0.628
Chronic obstructive pulmonary disease, *n* (%)	6 (8)	6 (8)	1.00
Hemoglobin, g/dL	11.5 ± 2.1	11.21 ± 2.2	0.319
Creatinine, mg/dL	1.2 ± 0.8	1.33 ± 1.2	0.537
LV ejection fraction, %	63.4 ± 6.1	63.1 ± 5.3	0.746
Left atrial volume index, mL/m^2^	43.2 ± 14.4	43.5 ± 14.7	0.895
Relative wall thickness,	0.49 ± 0.11	0.5 ± 0.14	0.719
LV mass index, g/m^2^	86.4 ± 29	90.3 ± 27.8	0.392
E-wave velocity, cm/s	90.7 ± 27.7	91.7 ± 31.7	0.834
E-DcT time, ms	281.5 ± 100.1	271.8 ± 91.3	0.531
E’ velocity septal, cm/s	5.1 ± 1.8394	5.5183 ± 2.077	0.185
E/e’ ratio septal	20.7 ± 11.4	19.5 ± 11.9	0.516
Tricuspid regurgitation velocity, m/s	2.5 ± 0.54	2.6 ± 0.43	0.882
Diastolic dysfunction (No/Yes/Indeterminate), *n* (%)	16 (21)/46 (58)/16 (21)	27 (35)/40 (51)/11 (14)	0.125
Death, *n* (%)	9 (12)	5 (6)	0.036
Cardiac Rehospitalization, *n* (%)	34 (44)	11 (14)	0.018
Composite, *n* (%)	39 (50)	13 (17)	0.014

The propensity score was calculated for matching of clinical parameters known to affect survival risk (age, diabetes, hypertension) as well as co-morbidities (anemia, defined by hemoglobin level, renal dysfunction defined by creatinine, and presence of chronic obstructive pulmonary disease), and absolute parameters of diastolic functions, namely left atrial volume index (LAVi), E/A ratio, e’ velocity from the septal mitral annular side, E/e’ ratio, and tricuspid regurgitation velocity.

## Data Availability

The data presented in this study are available on request from the corresponding author. The data are not publicly available due to privacy restrictions.
